# Appointment of Drs. S. D. Gross and Elisha Bartlett

**Published:** 1850-10

**Authors:** 


Drs. S. D. Gross and Elisha Bartlett have been appointed to the chairs of Surgery, and Institutes, and Practice of Medicine in the University of New York. Both these gentlemen held the same chairs in the University of Louisville, of which institution they were valued and efficient members. Dr. Gross, is well known as the author of an admirable and standard work on *Pathological Anatomy*, and as editor of *Liston's Elements of Surgery*. And Dr. Bartlett as author of a work *on Fevers*, most favorably received by the profession; and of an *Essay on the Philosophy of Medical Science*, and one on *Certainty in Medicine*.

The faculty of New York University have added to their strength in these appointments, and we congratulate them on having secured the services of such experienced collaborators.

